# Notes from Our Scrap-Book

**Published:** 1882-02

**Authors:** 


### NOTES FROM OUR SCRAP-BOOK.

The following notes are confined to symptoms connected with early and profuse menstruation. They are condensed and abbreviated so as only to give the most reliable and characteristic symptoms of a few of our best known drugs. The word *menses* is to be understood as beginning each sentence.

*Aconite.*—Profuse, long lasting; most often late and protracted, but scanty. Profuse, with nose bleed. (*Natr. S.;* Epistaxis before and during menses, *Sulphur.*)

*Agaricus.*—Profuse, with tearing, pressing pains in back and abdomen.

*Aloe.*—Early and profuse. Pains worse when standing.

*Ambra.*—Early and profuse; easy discharge of blood between periods, may be caused by a too long walk or hard stool. Left leg blue from swollen varices.

*Amm. carb.*—Premature and profuse, preceded by griping collicky pains; clotted, black blood; during menses, sleeplessness. (*Ign., Sepia, Natr. m.*) Diarrhœa before and during.

*Amm m.*—Early, with pain in abdomen and small of back, more profuse at night. Vomiting and diarrhœa. (*Verat. alb.*)

*Arsenic.*—Early, profuse, exhausting. (*Carbo veg.*, *Ipecac.*, *Sulph.*) Blood dark. (*Ant. c.*, *Cham.*, *Creas.*, *Crocus*, *Lach.*, *Nux v.*, *Puls.*, *Sulph.*)

*Asarum erop.*—Early, protracted, but not very profuse; with their appearance, has violent pain in small of back, which scarcely allows her to breathe.

*Bellad.*—Profuse (early and protracted rather than late), discharge of hot, bright-red blood. Color of blood changes, hence may sometimes be dark and clotted; bad smell; aggravation lying on back and from least jar; sacrum feels as if broken. (Back, *Camp.*, *Nux.*) Chilliness in back. (*Kali c.*) Coldness of back, evenings.

*Borax.*—Early and profuse, with colic and nausea.

*Bovista.*—Rather apt to be early and scanty than late or profuse. Every two weeks. (*Calc. phos.*, *Ipec.*, *Magn. c*, *Trillium.*) Much dark, clotted blood; only at night, or only in morning. (Worse when first rising, *Magn. c.*) Diarrhœa before (*Amm. c.*, *Silicea*, *Verat.*) and during menses, with bearing down towards genitals.

*Bromium.*—Early, profuse; bright-red (*Bell.*, *Ipec.*, *Hyos.*, *etc.*,) blood. Membranous shreds pass off. (Stringy, *Crocus.*) Passive metrorrhagia (*Helonias*), weakness and want of appetite.

*Bryonia.*—Early and profuse, blood dark; when suppressed, Epistaxis. (*Bell.*, *Ham.*, *Puls.*, *Silicea;* frequent nose bleed, *Acon.*, *Bry.*, *Puls.;* few drops, *Lach.*; black and stringy, *Crocus.*)

*Bufo.*—Early, with headache; chilliness; shifting pains; menses clotted, or fluid and pale.

*Cactus grand.*—Too soon; black, pitch-like; agonizing pains, worse evenings. Constrictive spasm in uterus. Menses when scanty cease on lying down. (Reverse, *Cyclam.*)

*Calcarea.*—Early, profuse and long; metrorrhagia from mental worry or excitement.

*Calc. phos.*—Early, blood bright with girls; late, blood dark (or first bright then dark) with women.

*Cannabis sat.*—Profuse, with dysuria.

*Cantharis.*—Early, profuse; blood black; breasts painful. (*Calc.*, *Conium*, *Helonias*, *Phytol.;* breasts hard, *Carbo an.*, *Viburnum;* burning, *Indigo*, *Lac. can.*)

*Carbo an.*—Early and long, but not profuse. (*Aconite;* scanty and long, *Ignatia.*) Delicate women with glandular troubles. Chilly; unsuccessful desire to eructate during menses. Flow weakens her so she can hardly speak. Blood dark.

*Carbo veg.*—Early and profuse; blood pale or thick (first pale, then dark and clotted, *Staph.*); corrosive, acrid smelling (acrid, *Amm. c., Carbolic acid, Creosotum, Silicea*).

*Causticum.*—Early, profuse; after ceasing, passes a little from time to time for days. Only during day. Discharge smells badly, and causes itching of vulva. (Itching, *Ambra, Carbo v., Conium, Creasote, Kali c., Lyc., Petrol., etc.*)

*Cham.*—Early, profuse, clotted blood, putrid smell; severe labor-like pains in uterus and tearing in legs. Blood dark, comes in sudden attacks, cold feet; worse on back.

*Chelidon.*—Profuse, protracted but late; pain under right scapula.

*China.*—Early and profuse; black clots, with spasm in chest and abdomen. Metrorrhagia, blood dark, with fainting and convulsions. Weakness from profuse discharge.

*Cocculus.*—Profuse and often; on standing it gushes out in a stream. (Worse standing, *Pallad., Sulph.*) Just before menstruation, is so weak she can scarcely stand. (Lassitude before menses, *Bell., Bromium, Cimicifuga, Nux m.*)

*Coffea.*—Profuse and long; coldness and stiffness of body; only in evening. Metrorrhagia, black lumps. Worse from motion. (*Bell., Bry., Ledum, Nux, Silicea.*)

NOTE.—Both *Aconite* and *Coffea* have fear of death, bright red face with fever in child-bed and other troubles. Aconite pains are unbearable, making patient furious and causing *foreboding* of death; *predicts* day; faints from pain. Inflammatory troubles with high fever, thirst and restless tossing about. *Coffea* pains are also unbearable but they cause patient to *cry* and *lament*, and *fear* death; very *excitable* and nervous.

*Creasote.*—Early, profuse, protracted; followed by an acrid smelling, bloody ichor; itching and biting of the parts, during the flow but much aggravated after it. (Ailments worse after menses, *Nux, Puls.*) Flow intermits, almost ceases and again commences. (*Nux, Sulph.*) Fainting and pulseless with metrorrhagic discharge, blood is dark, offensive and clotty.

*Crocus sat.*—Profuse and long, but regular as to time; blood is dark, clotty (*Calc. phos., Cham., China, Nux, Puls., Secale*), and stringy. Worse from slightest motion. (Only flows while moving, *Lilium t.*)

*Cyclamen.*—Profuse, frequent; flow is less when moving about (*Æsculus*); worse in evening when sitting. Metrorrhagia, with stupefaction of head and obscure vision. Dreads fresh air.

*Ferrum.*—Profuse, protracted and late (also, early); blood coagulated, light colored or dark, with labor-like pains; intermits two or more days, then returns; languor and weakness though discharge is not great. (*China*, from excessive discharge.) Hysterical symptoms after menses. (*Puls.* has cured hysteria with amenorrhœa.)

*Helonias.*—Profuse, frequent in women, weak from loss of blood. Flow passive, dark and offensive. Depressed, melancholy. Breasts swollen, nipples tender; cannot bear pressure of clothing. (*Conium, Thuja, Zinc.*)

*Hepar s. c.*—Profuse, early; metrorrhagia with chapped skin and rhagades of feet and hands.

*Hyos.*—Profuse, with convulsive trembling of hands and feet; headache, sweat, lock-jaw (*Nux*). Previous to menses much loud laughing; hysterical symptoms (after menses, *Ferr.*); sweat and nausea.

*Ignatia.*—Early and protracted, but rather scanty than profuse; black clots, putrid odor.

*Iodium.*—Early or late; if early are apt to be profuse; if late, short and scanty. Metrorrhagia, copious and violent. Iodium is one of those remedies which have irregular menstruation; at one month it comes too soon, next too late, so on. Other prominent ones are, *Berberis, Cocculus, Ferrum, Graphites, Nux m.* and *Sepia.*

*Ipecac.*—Early, profuse; metrorrhagia, blood bright-red, clotted; with oppressed breathing; nausea.

*Kali b.*—Early, rather scanty; early with vertigo, nausea and headache. Obstinate suppression of urine.

*Kali carb.*—Sometimes early, but most often late and scanty, often indicated in the delaying and difficult menses of young girls. (Also, *Aconite, Bry., Caust., Ferr. Graphites. Lyc., Natr. m., Petro., Puls., Sepia, Sulph.*)

*Lachesis.*—Generally scanty and short; but at the climaxis it is often indicated when menses are irregular and profuse; blood black, lumpy. Desires open air. (*Puls.*)

*Lauro.*—Early, long, profuse; discharge thin, with nightly pain in vertex.

*Ledum.*—Early, profuse; blood bright red; absence of vital heat.

*Lobelia.*—Early, profuse; violent pain in sacrum. Sense of great weight in genitals.

*Lycopod.*—Profuse, protracted and late; flow partly clotted, partly bright red or like serum; labor-like pains followed by swooning, (*Actea rac.*, *Cocc.*, *Glon.*, *Ign.*, *Lach.*, *Mosch.*, *Nux m.*, *Nux v.*, *Plumb.*, *Puls.*, *Sulph.*)

*Magn. m.*—Early, profuse or late, with pains in back when walking, in thighs when sitting; with hysterical symptoms; blood black, clotted; pale face, nervous excitement and debility.

*Merc. v.*—Profuse, with anxiety and colic: deep, sore pain in pelvis, dragging sensation in loins; abdomen feels weak as if it had to be held up.

*Millefolium.*—Profuse hemorrhages from over-exertion. (*Nitr. acid.*)

*Moschus.*—Early, profuse, with a drawing pain.

*Mur. ac.*—Early, profuse; dejection of spirits, silent, as if she would die. (Gloomy, etc., with menses, *Actea rac.*, *Ignat.*, *Natr. c.*, *Pulsat.*, *Sepia*, *Zinc.*) Genitals so sensitive cannot bear least pressure, not even a sheet. (*Lach.*, *Lilium*, *Pulsat.*)

*Natr. carb.*—Early and long; preceded by pain in neck and head, accompanied by tearing headache, bloated abdomen (mornings), relieved by diarrhœa: worse from music and during thunder-storm.

*Natr. m.*—Late, long but not very profuse; before menses is sad, anxious, with headache, palpitation, eructations; during, sad with headache and colic; after, headache.

*Nitr. ac.*—Early and profuse; aching from thighs; also early, irregular and scanty. During menses, cramp-like pain in abdomen, as if it would burst; brown offensive discharges; stitches in vagina when walking.

*Nux jugl.*—Early and profuse; black clots.

*Nux m.*—Irregular in time and quantity; profuse; dark flow, with aches and pains in back, limbs and abdomen; pain in small of back, as if a piece of wood lay across it. (*Rhus*, sensation as of a board strapped across forehead.)

*Nux vom.*—Early, protracted and profuse; flow, dark; patient irritable, nervous, faints easily; navel feels drawn in.

*Phosph.*—Early and profuse, or scanty and pale; weeps before menses. (*Cactus*, *Con.*, *Lyc.*, *Plat.*, *Puls.*) Gums swell before (*Baryta c.*) and during. (Swollen gums during, *Baryta*, *Merc.*, *Nitr. ac.*)

*Ph. ac.*—Early, long; pain in region of liver.

*Phytolac.*—Copious and frequent; mammæ painful. (*Calc.*, *Conium.*)

*Platina.*—Early and profuse but short; flow dark, preceded by spasms, bearing down, desire for stool (*Nux*); during the flow pains in abdomen and uterus, with melancholy. Metrorrhagia, body feels as if getting larger.

*Ratanhia.*—Early, profuse and long; pains in abdomen and small of back.

*Rhodo.*—Early, profuse; fever and headache.

*Rhus t.*—Early, profuse and long; flow light colored, acrid, with biting pain in vagina. (Aching in vagina, *Calc.*; burning, smarting pain, *Berberis.*)

*Sabina.*—Early, profuse; partly pale red fluid, partly clotted and offensive; flows in paroxysms; flow increased by motion but often ameliorated by walking.

*Sanguin.*—Regular; offensive smelling; bright red flow; clots like lumps of flesh; later, dark and less offensive flow; pain in head and eyes, ameliorated by vomiting.

*Secale.*—Profuse, protracted; tearing, cutting colic, cold extremities and cold sweat, weak; hemorrhage worse from slightest motion. Blood thin and black; black, lumpy, or brown fluid, and of disgusting smell.

*Sepia.*—Early and profuse; mania from profuse menstruation. Itching pain in vagina. (*Alum, Aurum, Conium, Creas.*) Bearing down pains cause oppression of breathing. (Oppression of breathing during menses, *Cactus, Calc., Cocc., Cupr., Graph., Ipec., Lyc., Puls., Zinc.*)

*Silicea.*—Late, long, but not very profuse; irregular, every two or three months. If early, scanty; if late, profuse. Flow strong smelling and acrid.

*Spongia.*—Soon and profuse; preceded by colic, backache, soreness in sacrum, craving in stomach, and palpitation. During menses awakes with suffocating spells.

*Stannum.*—Early and profuse, preceded by melancholy. (*Am. c., Calc., Caust., Con., Cyclam., Ferr., Lac. can., Lyc., Natr. m., Puls.*) Pain in malar bone.

*Staph.*—Irregular, late and profuse; first pale then dark and clotted. Sexual organs painful when sitting.

*Stram.*—Excessive flow, watery; loquacity, singing, praying, etc. Strong smell, as of semen. After menses, sobbing, whining.

*Sulphur.*—Generally short and late, but may be profuse with acid smell. Before menses, headache, nosebleed; during, nosebleed, rush of blood to head, weak, faint spells (at 11 A.M.). Flow thick, dark.

*Sulph. ac.*—Early, profuse; nightmare before menses. Climacteric spitting of blood.

*Verat. alb.*—Early, profuse; suppressed, with despair of salvation and spitting of blood. Dysmenorrhœa, with cold sweat, vomiting and diarrhœa.

*Zincum.*—Early, profuse; lumps of coagulated blood pass, mostly when walking; flow most profuse at night. Dysmenorrhœa, limbs heavy, violent drawing about knees, as if they would be twisted off. Pain in left ovarian region, better from pressure; entirely relieved during menstrual flow. (*Lachesis.*)

*Zingiber.*—Early and profuse; flow dark clotted; irritable, with exhausted look before menses come on. E. J. L.

